# On the Filler Materials of Metal Matrix Syntactic Foams

**DOI:** 10.3390/ma12122023

**Published:** 2019-06-24

**Authors:** Attila Szlancsik, Bálint Katona, Alexandra Kemény, Dóra Károly

**Affiliations:** 1 Department of Materials Science and Engineering, Faculty of Mechanical Engineering, Budapest University of Technology and Economics, Műegyetem rakpart 3., 1111 Budapest, Hungary; szlancsik@eik.bme.hu (A.S.); katona@eik.bme.hu (B.K.); kdora@eik.bme.hu (D.K.); 2 MTA–BME Lendület Composite Metal Foams Research Group, Műegyetem rakpart 3., 1111 Budapest, Hungary

**Keywords:** metal matrix syntactic foam, filler, hollow sphere, mechanical properties, microstructure

## Abstract

Metal matrix syntactic foams (MMSFs) are becoming increasingly relevant from the lightweight structural materials point of view. They are also used as energy absorbers and as core materials for sandwich structures. The mechanical properties of MMSFs are extensively influenced by the properties of their filler materials which are used to create and ensure the porosity inside the metal matrix. As the properties of fillers are of such importance in the case of MMSFs, in this paper three different filler materials: (i) ceramic hollow spheres (CHSs), (ii) metallic hollow spheres (MHSs) and (iii) lightweight expanded clay particles (LECAPs), have been investigated in numerous aspects. The investigations cover the microstructural features of the fillers and the basic mechanical properties of the fillers and the produced MMSFs as well. The microstructure was studied by optical and electron microscopy extended by energy-dispersive X-ray spectrometry, while the basic mechanical properties were mapped by standardized compression tests. It was found that in the terms of cost-awareness the LECAPs are the best fillers, because they are ~100 times cheaper than the CHSs or MHSs, but their mechanical properties can be compared to the aforementioned, relatively expensive filler materials and still exceed the properties of the most ‘conventional’ metallic foams.

## 1. Introduction

The field of lightweight materials has become one of the most investigated areas today. Every design method focuses on energy efficiency to gain the best price to value ratio, especially in mass production. One of the best way to increase the efficiency of a structure or machine is to reduce its weight. It can be done by using stronger materials which allow to design thinner structures, or by reducing the density while maintaining the strength of the material at a high level. If the choice is to reduce the density, closed-cell metallic foam is one of the best options. They are commonly made by injecting a gas or mixing a foaming agent into molten metal [1,2]. The main application fields are energy absorbers or sandwich panels and filled sections for structural applications [3,4]. Therefore, the ‘conventional’ open and closed cell foams have been widely studied; besides their basic mechanical properties, including elastic parameters [5], their behavior during cyclic load [6,7] or at high temperature have been in focus [4]. Metal matrix syntactic foams (MMSFs) are an upgraded version of ‘conventional’ closed-cell metallic foams. In these materials the porosity inside the matrix are created by a lightweight second phase or—as often referred to—filler material. Regarding the matrix it is most commonly an Al alloy, but Mg- [8], Fe- [9], Ti- [10] and even Zn alloys [11,12] can be found in the literature. The first filler materials to produce MMSFs were fly-ash microspheres. Fly-ash particles are by-products of fossil thermal power plants and in that way they are cheap and available in large quantities. On the other hand, their structure, shape and mechanical properties are not consistent and have large scatter [8,13,14].

Several types of filler materials have been investigated in the literature from which the most commonly applied grades belong to the group of ceramic hollow spheres. For example, Santa Maria et al. [15,16] produced Al_2_O_3_ hollow sphere-reinforced aluminum matrix syntactic foams. They determined the foams strength dependence on the filler size. The results of their work showed that the main property that increases the strength is the spheres’ wall thickness to diameter ratio. MMSFs produced with ceramic hollow spheres have outstanding mechanical properties such as wear [17], compressive [18,19] and tensile [20] properties, but the cost of these foams is relatively high because of the expensive filler. A version of ceramic hollow spheres is built up from ~40 wt.% Al_2_O_3_ and 60 wt.% SiO_2_, and these hollow spheres are somewhat cheaper, but have similar properties to pure Al_2_O_3_ made ceramic hollow spheres [21]. Orbulov et al. studied these materials [22] and explored their production possibilities [23,24], the effect of the fillers size [25] and the matrix material [26], the damage modes [27,28] and even their damping capability [29,30]. Li et al. [31] manufactured Al and Al-Mg matrix syntactic foams with glass cenospheres. They concluded that the filler material determined the failure behavior of the foam. Luong et al. [32] developed high performance SiC hollow spheres filled with MMSFs and characterized them in compressive load at quasi-static and at high strain rates. Szlancsik et al. [33,34,35] investigated the structure and mechanical properties of metallic hollow sphere reinforced aluminum matrix syntactic foams. Rabiei et al. [36,37] studied the production possibilities (liquid state infiltration and powder metallurgy) and the mechanical properties of iron hollow spheres filled with aluminum matrix composite foams. Another interesting application of the iron spheres is to create hollow sphere structures directly from them, without matrix material: Fiedler et al. [38] sintered metallic hollow spheres and measured their mechanical properties. The porosity of this structure has been reported to be as high as 86.7% and the achieved density was as low as 1.04 g cm^−3^. Taherishargh et al. [39,40,41] produced and tested aluminum foams filled with expanded perlite. The density of these foams was around 1.05 g cm^−3^ which is almost the same as of the previously mentioned hollow sphere structure, but the mechanical properties were at least four times higher, for example the plateau strength was ~8.5 MPa and ~40 MPa in the case of the hollow sphere structures and expanded perlite filled syntactic foams, respectively. Taherishargh et al. [42] also produced pumice-filled metal matrix syntactic foams. With this filler material they achieved a cost reduction in production, but the density of the foam was also higher (1.49 g cm^−3^).

The aforementioned filler materials were well characterized when they were used in MMSFs, even at elevated and cryogenic temperatures [43,44,45], but their individual properties have rarely been investigated, only the size and the composition of the particles are reported regularly. However, a few examples are existing in the literature about the production and investigation of the fillers themselves, that have to be mentioned. Cochran et al. [46] developed ceramic hollow spheres for electric applications. Song et al. [47] investigated the compression behavior of individual metallic hollow spheres. Yu et al. [48] dealt with the production of carbon hollow spheres. Orbulov and Májlinger [21] performed preliminary microstructural investigations on individual hollow spheres and on their connection to the matrix material. Ranjbar and Kuenzel summarized the properties of cenospheres [49].

The aim of this paper is to provide a connection between the features of the filler particles through their applicability as fillers in MMSFs to the mechanical properties of the produced MMSFs. According to this, our goals are (i) to measure five properties (particle density, loose bulk density, fracture strength, slope of the loading curve, price) which are relevant in terms of filler materials and (ii) to map the basic mechanical properties of the MMSFs produced by the application of the different fillers.

## 2. Materials and Methods

Three types of filler materials have been investigated with different methods, these are: ceramic hollow spheres (CHSs), metallic hollow spheres (MHSs) and lightweight expanded clay particles (LECAPs). From the CHSs and the LECAPs three and two different particle size groups were investigated, respectively. Their average diameter and notation are the following: Ø1.6 mm (CHS-1.6), Ø2.4 mm (CHS-2.4) and Ø7.0 mm (CHS-7.0) for the CHS grades and Ø3.0 mm (LECAP-3.0) and Ø9.0 mm (LECAP-9.0) for the LECAP grades, respectively. The chemical composition and the price of the fillers are detailed in Table 1. The price of the filler is also important, besides the physical properties in the cost-efficiency aspect. The prices were gathered based on short amount quotations and expressed in EUR per liter (EUR dm^−3^).

The surfaces of the fillers were examined by an Olympus SZX16 optical stereo microscope. With this method the average particle size was determined. From each filler type at least 100 individual particles were measured. To investigate the cross-section of the particles (with special focus on the walls’ structure in the case of hollow spheres) the particles were mounted into a resin commonly used in metallography and ground to the half of their average diameter on SiC grinding papers.

The density values—as one of the most important physical property of the filler materials—were also measured. For the individual densities of the particles, the particle volumes were calculated from the stereo microscopic measurements (geometry and volume), and the weight of the individual particles were measured by a Denver Instrument APX-200 scale (Denver Instrument Inc., Bohemia, NY, USA). The (bulk) density of the fillers was determined in loosely packed structure. This density is defined as the mass of the filler particles divided by the overall volume they occupy after vibrating the poured particles in the mold (including the particles volume and the volume between the individual particles).

Compression strength values of the filler materials were measured with an Instron 5965 universal testing machine (Instron, Norwood, MA, USA equipped with a 5 kN load cell. At least 50 particles were tested from each filler grade up to fracture one by one to obtain statistically evaluable results. The cross-head speed was set to 1 mm min^−1^.

Scanning electron microscopic (SEM) measurements were also performed with a Zeiss EVO MA10 microscope (Carl Zeiss AG, Oberkochen, Germany). The surfaces were investigated further in larger magnifications and energy-dispersive X-ray spectroscopy (EDS) (EDAX Inc., Mahwah, NJ, USA) was performed to determine the chemical composition of the materials.

After the comprehensive investigations of the individual filler particles, MMSFs were produced by low pressure liquid state infiltration method which has been described in detail elsewhere [35]. Briefly, the infiltration parameters were 400 kPa Ar gas pressure, 30 s holding time, 600 °C infiltration temperature. The volume fraction of the fillers was intended to be maintained at 64 vol% by pouring the particles into the mold and densifying them by vibrating the system. In this case, the theoretical volume fraction is about 64 vol% [50,51]. The matrix material was AlSi12, its chemical composition is listed in Table 2.

Line EDS measurement was performed on the MMSFs to determine the thickness of the boundary layer between the filler particles and the matrix material. It is essential to determine the connection and bonding between the matrix and the filler material. This boundary layer defines that how the filler particles work together with the matrix. If the bond is strong, the load transfers between the particles and the matrix is effective. If the bond is weaker the load transfer is less effective.

The mechanical properties of the produced MMSFs were measured on an MTS810 type servo-hydraulic universal testing machine (MTS Systems Corporation, Eden Prairie, MN, USA), between hardened, polished and lubricated plates in a two bar upsetting tool. The samples were cubic, with respect to the requirements of the ISO 13314:2011 standard [53] (dimensions of the sample should exceed 7–10 times the nominal diameter of the filler particles). The cross head speed was 0.1 mm s^−1^. At least five samples from each MMSF block were measured and the results were interpreted by the averages and by the scatter of the measurements.

## 3. Results and Discussion

### 3.1. Properties of the Filler Particles

Philosophically, two main features define whether the material is applicable as a filler material or not: (i) the surface of the filler should be solid and closed because if the molten metal fills the particle during the infiltration then the density reduction cannot be achieved; (ii) the density should be as low as possible (preferably below 1.0 g cm^−3^) because any other way and the manufactured foam’s density would be too high. These requirements are evidently extended by some others such as high strength, low price, good bonding to the matrix material etc. Every type of filler material that we investigated completely fulfilled these requirements.

**Figure 1:**
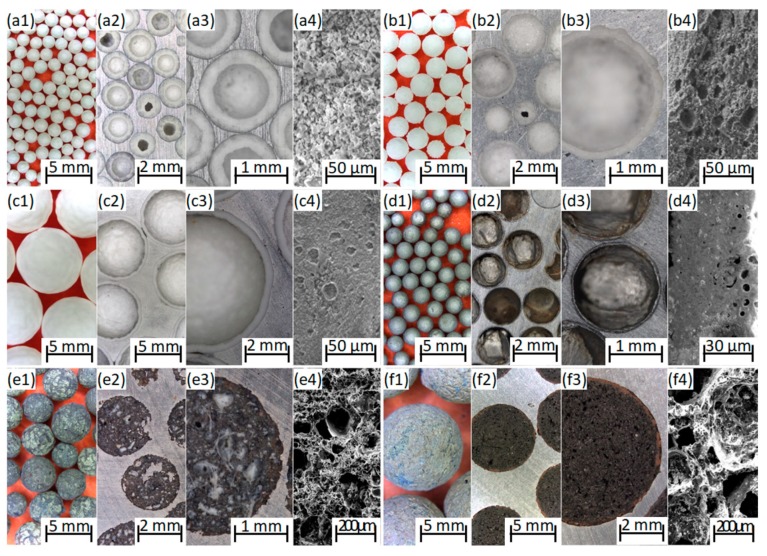
Geometrical features of the investigated fillers (**a**) CHS-1.6, (**b**) CHS-2.4, (**c**) CHS-7.0, (**d**) MHS, (**e**) LECAP-3.0 and (**f**) LECAP-9.0 along with the numberings of (**1**) macroscopic image of the sets of fillers, (**2**) low magnification image of the filler in AlSi12 matrix, (**3**) large magnification image of the filler in AlSi12 matrix and (**4**) scanning electron microscope (SEM) image of the fractured filler.

**Figure 2:**
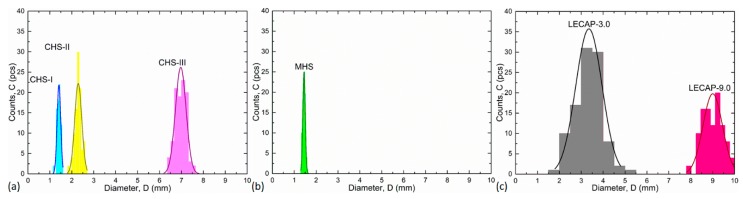
Size ranges and distributions for (**a**) CHS; (**b**) MHS and (**c**) LECAP filler grades.

Figure 1 shows the optical microscopic images of the filler materials. The CHSs have regular, spherical shape and completely closed surface but there are small spherical porosities in their walls near to the surface, especially in the case of CHS-2.4 and CHS-7.0 grades. These porosities are too small and closed for the aluminum alloy to infiltrate the spheres, but they can be useful in the boundary layer formation, if they are open to the surface. MHSs are consistent and have thin walls. The sphericality of MHSs is close to unity, the particles are almost perfect spheres. Some irregularities (thinner wall, sudden curvatures) can be observed along the periphery of the individual spheres. The LECAPs’ surface is solid and closed, but they have a lot of inconsistencies and inner porosities. The size of the pores inside the individual particles ranges between microns and a few millimeters, having strong influence on the mechanical properties of the particles. Figure 2 depicts the average particle sizes and the fitted Gaussian distributions. The LECAPs showed the largest scatter (the widest Gauss bell; please note the identical horizontal scaling). The reason behind this, is that the manufacturer provide that the particle sizes are in the range of 1–15 mm from which only the ranges of 1.5–5.5 mm and 8–10 mm were selected (sieved), based on the assumption that the most of the particles should fall in these ranges. The reason behind the selection of the size ranges was to have a similar and a higher average filler diameter group compared to the size distribution of CHS-2.4 and CHS-7.0 grade hollow spheres. The overlapping of CHS-1.6 and MHSs was also intended, with the purpose of comparing the fillers with almost identical size but very different material. Regarding the CHS fillers, the widening of the scatter of the hollow spheres can be observed clearly by the increment of the average diameter.

In the volume calculations, the diameter and the scatter have been calculated from the Gaussian distribution. The mean value is the mean value of the Gaussian fit, and the scatter is σ, which means that the 68% of particles are in this range. Table 3 contains the measured diameters and mass values, as well as the calculated volumes and densities. In general, the LECAPs and the CHSs were found to be the heaviest, but they ensured the highest strength values as well (see later). This trend was not confirmed by the CHS-1.6 grade, having the largest particle and loosest density (due to their quite thick wall), but low crush strength.

**Figure 3:**
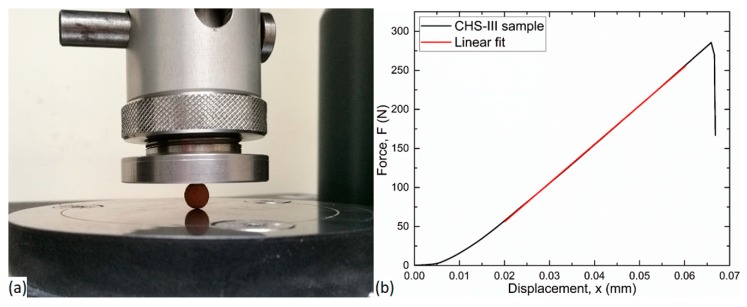
Measure compilation (**a**) and typical force–displacement curve with slope (**b**) of compression test of the individual hollow particle.

Figure 3a shows the measurement setup used for the compression test of the individual particles. A typical measured force–displacement curve can be seen in Figure 3b. As main characteristic properties, the fracture force (global maximum in the diagram) and the slope of the force–displacement curve were determined for each investigated particle.

Table 4 summarizes the compression data; every value was calculated from the results of at least 50 particles per filler grades.

**Figure 4:**
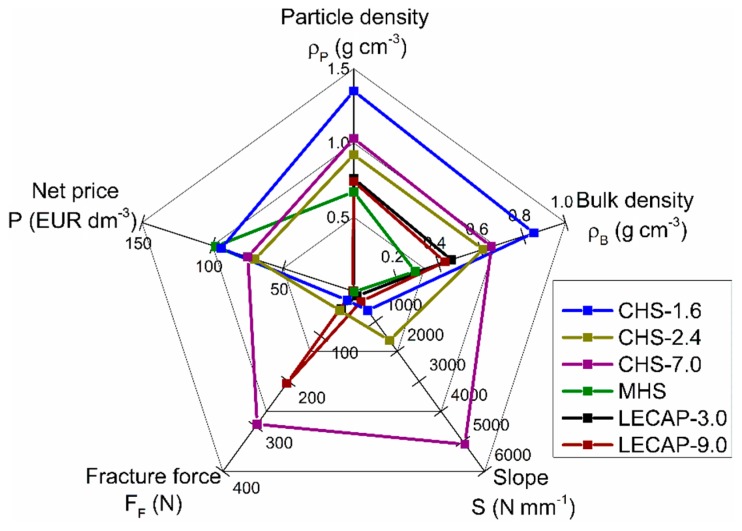
Radar plot of the most important properties of the investigated filler materials.

The CHS-7.0 grade filler had the highest fracture force which indicates that the syntactic foam made with this filler material should have the best mechanical properties (or at least the highest compressive strength); however, the scatter of the values was the highest, too. The LECAP-9.0s were close in strength to the CHS-7.0, they proved ~200 N fracture strength and with this result, found to be strong among the particles; however, a relatively large scatter (almost 60 N) was connected to this strength level. The reason for the large scatter can be found in the large-scale inner porosities (see Figure 1e,f) of the particles. The straight slopes of the force–displacement curves showed a linear relationship between the force and displacement values, but in the case of LECAPs and MHSs they were lower than expected. If the slope is high, the produced syntactic foam will have higher mechanical properties and especially high structural stiffness is expected. Based on the properties detailed and discussed above, it is possible to plot a radar graph for visual comparison of the filler materials’ performance (Figure 4). The plot helps to emphasize two grades, performing better compared to the others. In the mechanical properties aspect, the CHS-7.0 grade spheres are the best; however, their density and price are quite high. They can be used in applications where the strength and structural stiffness are extremely important and the price is neutral (aviation industry, space industry, defensive and military applications). LECAP-9.0s are also provided acceptable strength values with moderate densities and with extremely low price, therefore, they would be suitable for cheap and light structural materials subjected to moderate loads (for example in the mass production of automotive industry).

### 3.2. Properties of Metal Matrix Syntactic Foams (MMSFs)

**Figure 5:**
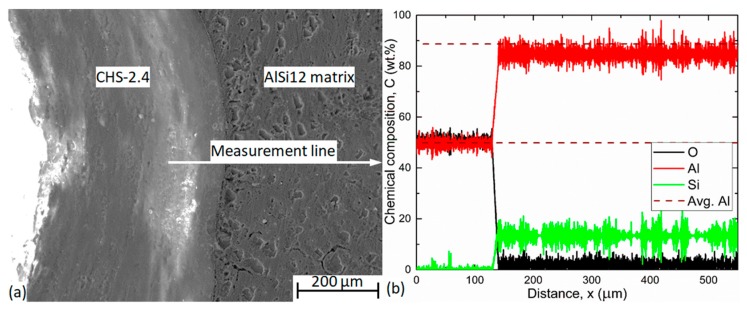
SEM picture (**a**) with the line used for the line energy-dispersive X-ray spectroscopy (EDS) measurement (red arrow) and (**b**) line EDS measurement result for the CHS-2.4 grade fillers.

Regarding the microstructure and the interface layer between the filler particles and the matrix material in the MMSFs, line EDS measurements were performed. The measurements started from the filler, intersected the interface layer perpendicularly and finally ended in the AlSi12 matrix material quite far (more than 300 μm) from the investigated filler particle. A typical line EDS SEM image and the results for AlSi12 matrix and CHS-2.4 grade ceramic hollow sphere filler are shown in Figure 5 as an example. In general, the tracked and recorded chemical elements were: O, Al, Si and Fe as the main chemical constituents of the matrix and the fillers. In the filler and in the matrix, the compositions refer to the chemical composition of the constituents, respectively. In the filler, Al and O could be found due to the dominant Al_2_O_3_ composition of the CHS-2.4 filler and Si was recognized within the scatter of the measurement. In the matrix the previously measured ~88 wt.% Al and ~12 wt.% Si were confirmed and minimal O content was detected (within the scatter of the measurement) due to the natural oxidation of the matrix. In order to define the interface layer thickness (that is in connection to the bond strength between the filler and the matrix), first the average aluminum percent was determined both in the matrix and in the filler (dark red dashed lines in Figure 5b). Subsequently, a straight line was fitted on the Al results in the interface region. The thickness of the interface layer was calculated as the horizontal distance between the intersection points of the fitted and average lines (Figure 5b). The boundary layer thicknesses for each filler material are listed in Table 5.

The results show that the boundary layer thickness was almost the same for each filler material. This phenomenon can be explained in the case of ceramic fillers that consist of alumina and silica. The molten Al alloy would react with the silica content of these materials. However, this diffusional reaction (propelled by the Si content mismatch of the particles and the matrix) is suppressed by the high Si content of the matrix and the short holding time during production [15,16]. In summary, this means that this property is not relevant in order to choose from the different type of materials, at least in the case of the investigated MMSFs.

The basic mechanical properties of the MMSFs can be interpreted through the characteristic properties of the materials. Hereby, the compressive strength (σ_C_ (MPa), representing the overall load-bearing capacity of the MSSFs), the fracture strain (ε_C_ (%), the strain value at the compressive strength), the structural stiffness (S (MPa), the slope of the initial, linear part of the MMSFs), the plateau strength (σ_PL_ (MPa), the strength level of mechanical energy absorption), and the absorbed mechanical energy values (W_50_, (J cm^−3^), up to 50% compressive strain) are considered. These values can be determined according to the prescriptions of the ruling ISO 13314:2011 standard [53]. The measured values are listed in Table 6, along with the relative densities (the actual density of the MMSF divided by the density of the matrix) of the investigated MMSFs.

**Figure 6:**
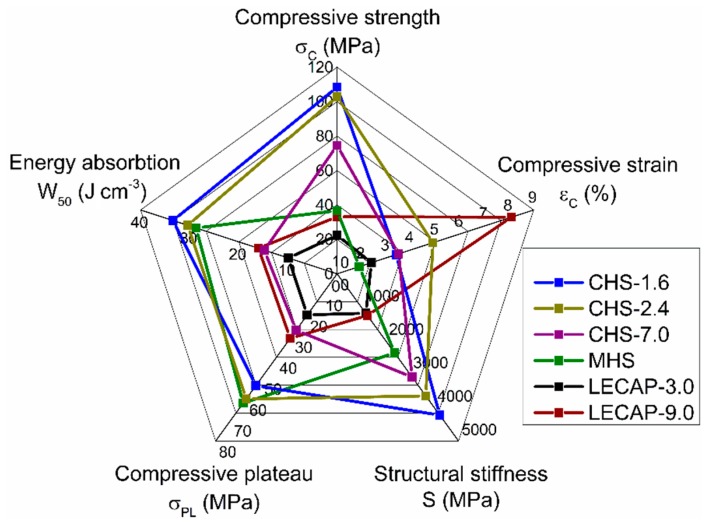
Radar plot of the most important properties of the foams produced with the investigated filler materials.

For better visualization, the radar plot of the average values summarized in Table 6 is shown in Figure 6. The smaller (1.6 mm and 2.4 mm) CHS grades granted higher strength, stiffness and energy absorption values with acceptable compressive strains, and therefore they are the best choices for high-performance structural applications. The CHS-7.0 grade fillers performed surprisingly bad when built in MMSFs, compared to their standalone properties, when tested individually. The reason is in the different loads (in MMSFs, the load is distributed, in standalone tests, the load was concentrated). In the case of MHS the plateau strength was outstanding and connected to a good energy absorption capacity, while the compressive strength and the structural stiffness were moderate and the compressive strain was low. Therefore, these MMSFs can be good candidates for collision damping and protective applications, where a high amount of mechanical energy should be absorbed. Finally, the LECAP filled MMSFs showed the lowest mechanical properties, but were still stronger than ‘conventional’ closed cell metallic foams. However, LECAP-9.0 filled MMSFs were comparable to the CHS-7.0 grade, at least in the aspect of plateau strength and energy absorption. This fact becomes more interesting, when the costs of CHS-7.0 and LECAP-9.0 are compared (75 EUR/L versus 0.68 EUR/L, respectively). Therefore, LECAP-9.0 filled MMSFs can be considered as proper candidates for large volume, cheap, energy-absorbing applications.

## 4. Conclusions

Three different grades of filler materials were investigated to determine which has the best properties in terms of producing metal matrix syntactic foams (MMSFs). From the lightweight expanded clay particles (LECAPs) and the ceramic hollow spheres (CHSs), two and three different average diameter sets were measured, respectively. From the above investigations and results, the following conclusions can be drawn focusing on the suggested application fields of the different filler grades:

- From the mechanical properties point of few, the individual CHS-7.0 particles proved to have the highest performance, but in MMSFs, these fillers showed lower mechanical strength values compared to the smaller CHSs. CHS-filled MMSFs can be applied as high-performance, lightweight structural parts.
- Metallic hollow spheres (MHSs) showed as high plateau strength and energy absorption capability as the high-performance CHSs, but without compressive strength and structural stiffness that was too high. All of these features are connected to low compressive strains; therefore, MHSs-filled MMSFs are excellent candidates for energy-absorbing applications, such as protective devices and collision dampers.
- The mechanical properties of LECAP0filled MMSFs can be considered low, but still higher compared to ‘conventional’ closed cell metallic foams. Considering the low price of LECAPs, these MMSFs can be considered for cheap high energy-absorbing capability applications in large volumes, such as in the case of building protection.

In summary, the investigation of the different filler particles (individually and in built-in condition) revealed that, by the proper selection of the constituents, the expected properties of the MMSFs can be tailored to the requirements of a given application.

## Figures and Tables

**Table 1:** Chemical composition (in wt.%) and price (in EUR dm^−3^) of the filler materials.

Filler	O	Al	Si	Fe	Other	Price
CHS-1.6	59.85	21.00	19.15	-	-	93.75
CHS-2.4	49.19	46.82	0.94	-	3.05	70
CHS-7.0	49.18	48.99	1.10	-	0.73	75
MHS	4.40	-	-	95.89	4.11	98
LECAP-3.0	55.40	32.55	7.64	2.41	2.00	0.13
LECAP-9.0	49.10	12.78	22.84	7.70	7.58	0.68

**Table 2:** Chemical composition of AlSi12 (wt.%).

Main Components (wt.%)	Al	Si	Fe	Other	Closest Standard Equivalent [52]
AlSi12	86.0	12.8	0.1	1.1	A413.0

**Table 3:** Diameter, volume, mass, density and wall thickness values of the filler materials.

Filler	Diameter (mm)	Volume (mm^3^)	Mass (g)	Particle Density (g cm^−3^)	Loose Bulk Density (g cm^−3^)	Wall Thickness (μm)
CHS-1.6	1.41 ± 0.08	1.49 ± 0.26	0.0020 ± 0.0003	1.35 ± 0.17	0.85 ± 0.05	60 ± 1.7
CHS-2.4	2.29 ± 0.16	6.41 ± 1.32	0.0068 ± 0.0013	0.92 ± 0.05	0.61 ± 0.04	24 ± 0.5
CHS-7.0	6.97 ± 0.25	177.90 ± 18.54	0.1754 ± 0.0149	1.03 ± 0.05	0.65 ± 0.03	21 ± 1.1
MHS	1.45 ± 0.05	1.59 ± 0.19	0.0023 ± 0.0004	0.67 ± 0.09	0.29 ± 0.05	23 ± 0.6
LECAP-3.0	3.24 ± 0.65	19.47 ± 10.73	0.0141 ± 0.0074	0.76 ± 0.20	0.46 ± 0.02	-
LECAP-9.0	9.00 ± 0.45	384.89 ± 56.77	0.2862 ± 0.0469	0.74 ± 0.05	0.43 ± 0.02	-

**Table 4:** Mechanical properties of individual filler particles.

Filler	Fracture Force (N)	Slope (N mm^−1^)
CHS-1.6	19.8 ± 4.6	634 ± 170
CHS-2.4	42.3 ± 24.6	1637 ± 717
CHS-7.0	295.3 ± 39.0	5096 ± 459
MHS	19.6 ± 7.4	15 ± 7
LECAP-3.0	38.1 ± 11.8	153 ± 142
LECAP-9.0	204.5 ± 59.1	337 ± 163

**Table 5:** Boundary layer thickness values between AlSi12 matrix and the filler materials.

Filler	Boundary Layer Thickness (µm)
CHS-1.6	8.23 ± 1.79
CHS-2.4	6.29 ± 3.07
CHS-7.0	6.25 ± 1.75
MHS	6.26 ± 1.49
LECAP-3.0	5.52 ± 1.24
LECAP-9.0	6.15 ± 1.86

**Table 6:** The relative density and mechanical properties of the produced metal matrix syntactic foams (MMSFs).

Filler	ρ_rel_ (-)	σ_C_ (MPa)	ε_C_ (%)	S (MPa)	σ_PL_ (MPa)	W_50_, (J cm^−3^)
CHS-1.6	0.69 ± 0.01	108.4 ± 7.2	3.4 ± 0.3	4229 ± 191	53.3 ± 9.5	33.4 ± 1.7
CHS-2.4	0.61± 0.01	102.9 ± 4.0	4.9 ± 0.2	3655 ± 220	59.9 ± 2.6	30.4 ± 0.9
CHS-7.0	0.61± 0.01	74.6 ± 5.0	3.5 ± 0.5	3089 ± 252	27.0 ± 4.4	14.8 ± 1.2
MHS	0.52± 0.03	36.7 ± 4.8	1.9 ± 0.5	2369 ± 52	61.9 ± 5.2	28.7 ± 1.7
LECAP-3.0	0.48± 0.01	22.4 ± 1.7	2.4 ± 0.3	1180 ± 123	19.7 ± 2.2	9.9 ± 0.9
LECAP-9.0	0.57± 0.06	33.1 ± 3.7	8.1 ± 2.8	1249 ± 97	30.9 ± 5.8	16.0 ± 2.9
